# A *Myo6* Mutation Destroys Coordination between the Myosin Heads, Revealing New Functions of Myosin VI in the Stereocilia of Mammalian Inner Ear Hair Cells

**DOI:** 10.1371/journal.pgen.1000207

**Published:** 2008-10-03

**Authors:** Ronna Hertzano, Ella Shalit, Agnieszka K. Rzadzinska, Amiel A. Dror, Lin Song, Uri Ron, Joshua T. Tan, Alina Starovolsky Shitrit, Helmut Fuchs, Tama Hasson, Nir Ben-Tal, H. Lee Sweeney, Martin Hrabe de Angelis, Karen P. Steel, Karen B. Avraham

**Affiliations:** 1 Department of Human Molecular Genetics and Biochemistry, Tel Aviv University, Tel Aviv, Israel; 2 Wellcome Trust Sanger Institute, Wellcome Trust Genome Campus, Hinxton, Cambridge, United Kingdom; 3 Department of Physiology, University of Pennsylvania School of Medicine, Philadelphia, Pennsylvania, United States of America; 4 Department of Biochemistry, George S. Wise Faculty of Life Sciences, Tel Aviv University, Tel Aviv, Israel; 5 Section of Cell and Developmental Biology, University of California San Diego, La Jolla, California, United States of America; 6 Helmholtz Zentrum München, German Research Center for Environmental Health, Institute of Experimental Genetics, Neuherberg, Germany; 7 Technical University of Munich, Munich, Germany; Harvard Medical School, United States of America

## Abstract

Myosin VI, found in organisms from *Caenorhabditis elegans* to humans, is essential for auditory and vestibular function in mammals, since genetic mutations lead to hearing impairment and vestibular dysfunction in both humans and mice. Here, we show that a missense mutation in this molecular motor in an ENU-generated mouse model, Tailchaser, disrupts myosin VI function. Structural changes in the Tailchaser hair bundles include mislocalization of the kinocilia and branching of stereocilia. Transfection of GFP-labeled myosin VI into epithelial cells and delivery of endocytic vesicles to the early endosome revealed that the mutant phenotype displays disrupted motor function. The actin-activated ATPase rates measured for the D179Y mutation are decreased, and indicate loss of coordination of the myosin VI heads or ‘gating’ in the dimer form. Proper coordination is required for walking processively along, or anchoring to, actin filaments, and is apparently destroyed by the proximity of the mutation to the nucleotide-binding pocket. This loss of myosin VI function may not allow myosin VI to transport its cargoes appropriately at the base and within the stereocilia, or to anchor the membrane of stereocilia to actin filaments via its cargos, both of which lead to structural changes in the stereocilia of myosin VI–impaired hair cells, and ultimately leading to deafness.

## Author Summary

Human deafness is extremely heterogeneous, with mutations in over 50 genes known to be associated with this common form of sensory loss. Among them, mutations in five myosins are associated with human hereditary hearing impairment, demonstrating that this family of proteins is essential for the proper function of the inner ear. Myosins, motor proteins found in eukaryotic cells, are responsible for actin-based motility. Composed of a motor domain and a tail, the former binds filamentous actin and uses ATP hydrolysis to generate force and move along the filaments, while the latter binds to cargos in the cell. Myosin VI is unique among myosins due to its movement along actin towards the minus or pointed end, rather than the positive or barbed end. Mutations in this myosin are associated with human deafness. Much of our information regarding myosin VI comes from studies in cell culture or mouse mutants with mutations leading to deafness. Here, we describe a deaf mouse mutant, Tailchaser, with a mutation in myosin VI. Our data describe new functions for myosin VI in the hair cells of the inner ear, showing how alterations in this motor can lead to a human sensory disorder.

## Introduction

The molecular motor myosin VI is known to function as either an actin-based anchor or as a transporter, based on biochemical, biophysical and cell biological studies (reviewed in [1]). This protein moves along actin toward the minus end, in the opposite direction to all other characterized myosins to date [2]. Myosin VI achieves ‘gating’ or coordination of movement along actin by first having the rear head of the dimer strongly bound to actin, while blocking the lead head from binding ATP and thus continuing through its ATPase cycle until the rear head is released [3]. This is achieved by a unique insert near the myosin transducer region that facilitates communication between the actin interface, myosin lever arm, and nucleotide-binding elements of the motor domain.

Myosin VI plays an essential role in *Drosophila*, where it was originally discovered [4],[5], as well as in *C. elegans* [6], mice [7], and zebrafish [8]. Mutations in these organisms have confirmed myosin VI function in sperm individualization during spermatogenesis by remodeling of the plasma membrane, unequal portioning of organelle and cytoskeletal components, basal protein targeting and spindle orientation in mitotic neuroblasts, and regulation of actin-based interactions with the plasma membrane. In mammalian cells, myosin VI appears to be involved in clathrin-mediated endocytosis and maintenance of Golgi morphology and protein secretion, as well as movement and clustering of receptors and vesicle scission of endocytic vesicles [9]–[11]. Most compelling, mutations in the myosin VI gene are associated with a dominantly inherited form of human hearing loss, DFNA22 [12], a recessively inherited form of human deafness, DFNB37, and hypertrophic cardiomyopathy [13],[14]. Interactions with proteins have been defined for myosin VI with GIPC1 (GAIP-interacting protein, C-terminus) that recruits myosin VI to uncoated vesicles [15], SAP97 [16] and Dab2 [17] that recruit myosin VI to clathrin-coated pits and vesicles, and optineurin, required for myosin VI localization at the Golgi complex [18]. Most recently, TRAF6-binding protein (T6BP) and nuclear dot protein 52 (NDP52) interactions with myosin VI have implicated a role for this protein in membrane trafficking pathways with cell adhesion and cytokine-dependent cell signaling [19]. Myosin VI and vinculin were recently shown to interact, suggesting that myosin VI acts as an actin-based anchor to facilitate vinculin's link with cadherin complexes on actin filaments in epithelial cells [20]. Though by convention a cytoplasmic protein, myosin VI has been identified in the nucleus of mammalian cells, where it modulates the RNA polymerase II-dependent transcription of active genes [21].

In the inner ear, though myosin VI is clearly essential, its exact role remains a mystery. Myosin VI is one of the earliest hair cell-specific proteins, expressed in the mouse cochlea as early as embryonic day (E)13 [22]. In the inner and outer hair cells of the organ of Corti, myosin VI was suggested to serve as an anchor, maintaining the structure of the stereocilia [23]. Most of our information regarding protein function in the inner ear has come from the study of mouse mutants with mutations in genes encoding these proteins. Over 190 deaf and circling mutants exist in which the mutant gene is known and in 46 of these cases, they serve as corresponding models for human hereditary hearing loss (The Jackson Laboratory's Hereditary Hearing Impairment in Mice database, http://hearingimpairment.jax.org/master_table.html; [24]). The mutants include those that arose spontaneously, radiation-induced mutants, mutants produced by gene-targeted mutagenesis and *N*-ethyl-*N*-nitrosourea (ENU)-generated mutants (reviewed in [25]). The mouse Snell's waltzer mutant, a spontaneous mutation that arose in a colony at the Jackson Laboratory in 1966, was found to contain mutations in myosin VI (*Myo6*) approximately 30 years later [7]. Instrumental for this search was a radiation-induced mutant, *se^sv^*. Research using mouse models for deafness have emphasized the need for multiple alleles, since each mutation may lead to new phenotypic descriptions, allowing for elucidation of new functions for a given protein. Tailchaser, an ENU-generated dominant mouse mutant that arose from a large-scale mutagenesis screen [26],[27], was identified as a deaf and circling mouse mutant and shown to display a gradual deterioration of both hearing and balance function, similar to forms of dominant nonsyndromic deafness in humans. A scanning electron microscopy (SEM) study revealed that *Tlc* stereocilia bundles fail to form the characteristic V-shape pattern at birth, and by adulthood, hair bundles are severely disorganized and eventually degenerate. We have now identified a missense mutation in myosin VI in *Tlc* and revealed new insights into myosin VI function with this new *Myo6* allele.

## Results

### Mapping of the *Tlc* Mutation to Chromosome 9

**Figure 1:**
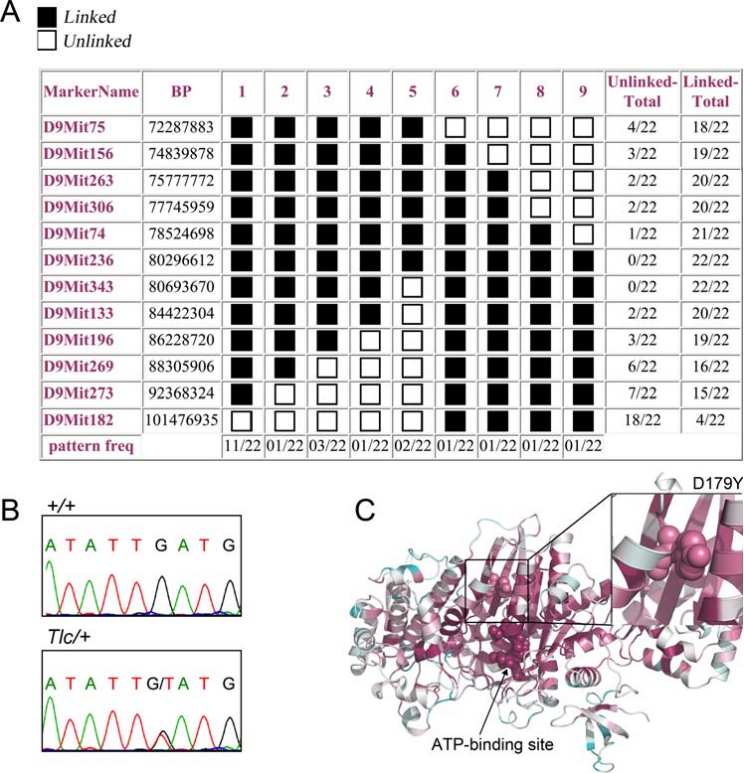
The Tailchaser mutation in myosin VI. (A) Pattern program output of the results of 10 markers located between *D9Mit75* and *D9Mit182*, tested on DNAs derived from 22 *Tlc* N2 mice from both the C3H and C57 backcrosses that showed recombination between these 2 markers. The *Tlc* mutation was determined to lie between markers *D9Mit74* to *D9Mit133*, with full linkage between markers *D9Mit236* to *D9Mit343*. The numbers across the top represent the different haplotypes. The black box (linked) demonstrates mice homozygous for the C3H backcross and heterozygous for the C57 backcross; the white box (unlinked) demonstrates mice heterozygous for the C3H backcross and homozygous for C57 backcross. (B) Sequencing of *Myo6* showing a G694T transversion in exon 6. Control cDNA (*+/+*) shows a single G peak and heterozygote cDNA (*Tlc/+*) shows boths two peaks of G and T. (C) Ribbon representation of the protein structure of a *Nest*-modeled mouse myosin VI with coloring according to the ConSurf scheme. ConSurf assigns amino acid conservation grades in the range of 1–9, where 9 is maximal conservation and 1 is maximal variability. The D179 residue, shown using a balls-and-sticks representation, was assigned a score of 8. For comparison, the ATP-binding domain, which is the most important region of the protein (also in balls and sticks), was assigned a score of 9.

Linkage for the *Tlc* mutation was found to chromosome 9 between markers *D9Mit104* and *D9Mit182* in a genome scan of 21 randomly selected N2 *Tlc/+* mice from a [C3HeB/FeJ-*Tlc/+*×C57BL/6J]×C3HeB/FeJ backcross. There was no evidence of linkage to any of the other autosomal chromosomes. Linkage to sex chromosomes was excluded by analyzing the mating data. Extending the analysis to a total of 84 N2 mutant mice and using an additional marker within the identified linkage interval confirmed linkage to mouse chromosome 9 to a region of 29 Mbp between *D9Mit75* and *D9Mit182*. Genotyping of additional polymorphic markers on all 183 available N2 mutant mice revealed 22 mice with recombinations in the region of the mutation, narrowing the region to 6 Mbp between *D9Mit74* and *D9Mit133* (Figure 1A). This linkage interval contained 28 known RefSeq genes (http://genome.ucsc.edu). Previously, we reported that the *Tlc* mutation was most likely localized to chromosome 2, although there were some inconsistent genotypes [27]. Analysis to further define the region demonstrated that C57 backcrossed N2 mutants (from a [C3HeB/FeJ-*Tlc/+*×C57BL/6J]F1×C57BL/6J backcross) presented a milder mutant phenotype when compared to the N2 progeny of the C3H backcross mutants, suggesting reduced penetrance. In the original analysis, only C57 backcrossed N2 mutants were analyzed, that may have led to erroneous phenotyping and subsequently incorrect matings and therefore mistaken localization.

**Figure 2:**
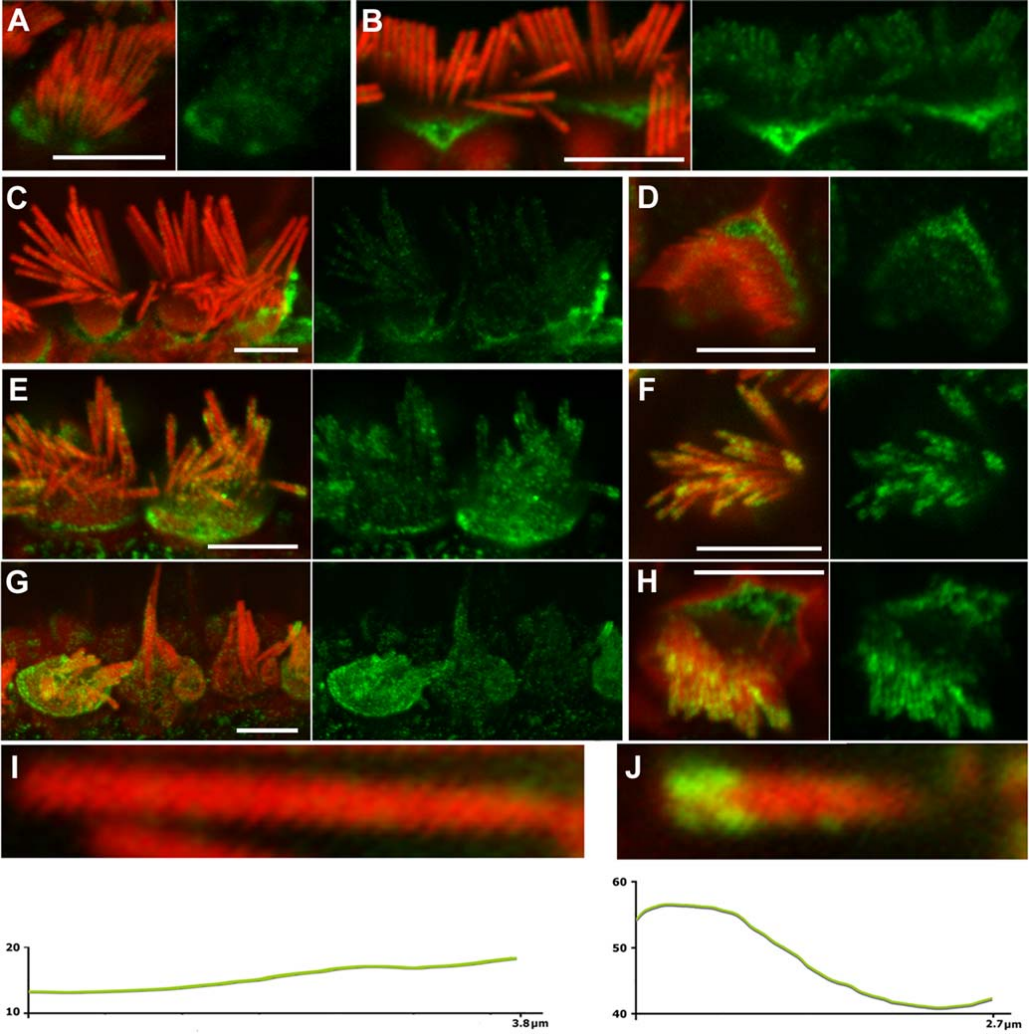
Myosin VI expression and localization in Tailchaser hair cells. (A–J) Expression of myosin VI (green) in wild type (A–D and I) and *Tlc/Tlc* (E–H and J) cochlear whole mount preparations, with filamentous actin labeled with rhodamine/phalloidin (red). Merged and corresponding green channel images are shown next to each other. Myosin VI-specific immunostaining can be detected in wild type and *Tlc/Tlc* stereocilia on inner (A–C, E, G, I, J) and outer (D, F, H) hair cells at P6 (A) and P70 (B–J) and appears to be outside of the stereocilia actin core. In wild type in the apical turn, myosin VI specific immunostaining is evenly distributed along the length of the stereocilium, with an increase at the base (A–D, I), while *Tlc/Tlc* stereocilia often show concentration of myosin VI-specific staining near stereocilial tips (E–H and J). In the middle turn of the cochlea, where hair cell degeneration was less pronounced, the immunofluorescence pattern was the same in inner (E) and outer (F) hair cells. Fused and elongated inner hair cells stereocilia in the basal turns of the cochlea showed a more diffuse pattern of myosin VI immunostaining (G). Green pixel intensity profiles of individual wild type (I) and *Tlc/Tlc* (J) stereocilia were obtained from images acquired at the same settings of the confocal microscope. Y axis, green pixel intensities on grey scale from 0 to 256; X, stereocilium length measured from stereocilium tip toward stereocilium base. Scale bars: A–H–5 µm.

### Identification of the Myosin VI *Tlc* Mutation

The unconventional myosin VI (gene symbol, *Myo6*) is localized in the center of the chromosome 9 non-recombinant interval. Myosin VI is an actin-based molecular motor that has been previously shown to underlie hereditary hearing loss in both human and mice (see Introduction). The *Myo6* gene encodes a 1265 amino acid protein (140 kD) that consists of an N-terminal motor domain, a calmodulin interacting neck domain and a C-terminal tail domain that is necessary for binding cargo and forming myosin VI homodimers (reviewed in [1]). Direct sequencing of *Myo6* cDNA extracted and amplified from *Tlc/+* brains revealed a c.G694T transversion in a region that corresponds to exon 6 of the *Myo6* gene (NM_008662) (Figure 1B). This mutation was confirmed by reverse sequencing of new mutant and wild type cDNA and direct sequencing of *Tlc/+*, *Tlc/Tlc*, wild type, *sv/sv*, C3H, C57 and *Spretus* genomic DNA (data not shown). The mutation was identified only in *Tlc* mutant mice and in none of the other DNA samples tested. A restriction digestion assay of PCR-amplified *Myo6* genomic DNA was tailored to identify the mutation as an alternative method of genotyping to screen all mice (see Materials and Methods). The G694T mutation is predicted to result in an aspartic acid to tyrosine amino acid substitution at position 179 of the myosin VI protein (p.D179Y). The conservation pattern of myosin VI was analyzed using ConSurf, which uses the Rate4Site algorithm for estimating the evolutionary rates at each amino acid site (Figure 1C) [28]. Aspartic acid in position 179 is highly conserved. Noteworthy is the fact that in the positions aligned with the D179 residue in the multiple sequence alignment (MSA), none of the 77 sequences, found in a hidden Markov model (HMM) search based on similarity to known myosin VI sequences, harbor an aromatic amino acid.

### Myosin VI Is Concentrated at the Tips of Tailchaser Stereocilia

The expression pattern of myosin VI in *Tlc* mutant inner ears was evaluated by immunohistochemistry with an antibody that detects the tail of myosin VI, and phalloidin, which labels filamentous actin. The specificity of the antibody used was validated by Hasson and colleagues [29] and was confirmed using auditory sensory epithelia of Snell's waltzer mice (Figure S2). Previously, myosin VI staining was observed in the cell body, the cuticular plate and pericuticular necklace of hair cells [29] and between the actin core and plasma membrane of stereocilia [30]. We confirmed this general expression pattern in wild type mice, in which specific immunostaining was evenly distributed along the length of control stereocilia (Figure 2A–D, I), with an increase at the base. Myosin VI expression was also observed in hair cells from *Tlc/Tlc* mutants, but with a significant difference in distribution in adults (P70), with stereocilia often showing enhanced myosin VI staining in the upper third portion (Figure 2E–H, J). The same staining pattern of myosin VI was observed in all turns of the cochlea despite much more pronounced hair cell degeneration and hair cell death in the basal turn. Only fused stereocilia forming giant protrusions showed a more diffused staining pattern. A comparison of green pixel intensity profiles revealed that the intensity of myosin VI specific staining was higher in *Tlc/Tlc* stereocilia (53.81±30.25, n = 95; *t*-test: 4.7×10^−16^) than in control stereocilia (15.81±7.93, n = 53).

### Hair Bundles of Tailchaser Mutants Are Severely Disorganized as Early as P1

**Figure 3:**
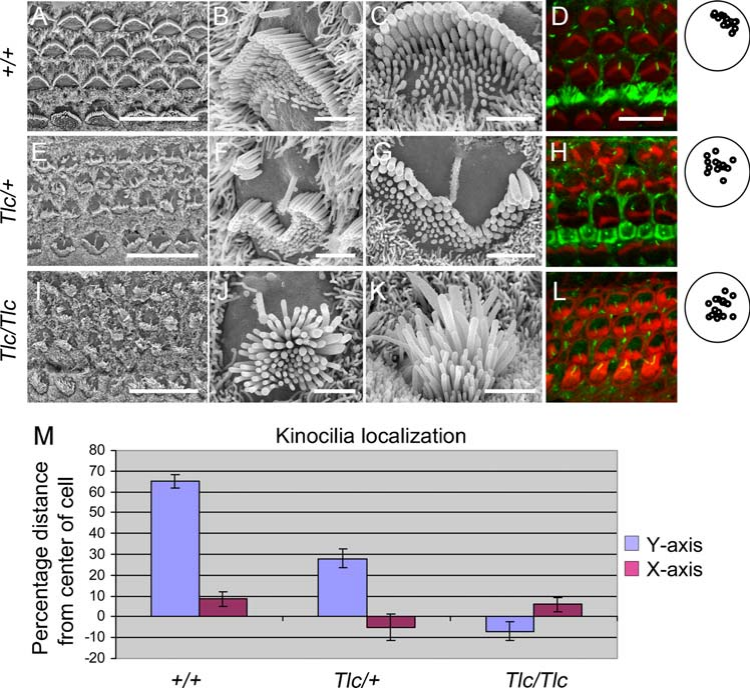
The kinocilia is mislocalized in Tailchaser hair cells. (A–I) Scanning electron microscopy images showing auditory sensory epithelia of wild type (A–C), *Tlc/+* (D–F) and *Tlc/Tlc* (G–I) mice at P0. Low magnification images (A, D and G) show that the characteristic arrangement of three rows of outer hair cells and a single row of inner hair cells is maintained in Tailchaser mutants. In wild type animals, outer (B) and inner (C) hair cell stereocilia form already highly organized bundles with kinocilium present at the center of the apical plane (arrow). Tailchaser mutants, however, show highly disorganized and misshaped stereocilia bundles on outer (E and H) and inner hair cells (F and I). Also, the position of the kinocilium was highly variable in Tailchaser mutants. Note remnants of a staircase-like stereocilia arrangement in *Tlc/+* hair cell. (D, H, L) Confocal microscopy images showing kinocilium position in wild type (D), *Tlc/+* (H) and *Tlc/Tlc* (L) at P0. Actin in stereocilia bundles was labeled with phalloidin (red). The kinocilia are labeled with an antibody that detects acetylated tubulin (green). A summary of positions of the kinocilia in outer hair cells demonstrates that kinocilia are more centrally located in mutants. (M) Kinocilium localization is represented as percentage of the distance from the center of the cell to its perimeter both in the Y (modiolar-striolar) and in the X (apical-basal) axes. Scale bars: A, D and G–15 µm; B, C, E, F, H, I–2 µm; D, H, L–5 µm.

In wild type mice, at E18.5, all hair cells are already present and their hair bundles are aligned with a slight degree of hair bundle disorientation, as demonstrated by SEM. Both inner and outer hair cell stereocilary bundles in all turns of the cochlea show a staircase-like organization with rows of stereocilia of graded height (data not shown). By P1, the stereocilia of the second row have a larger diameter than those from other rows on each cell and their tips are pointed in middle and basal turns [31]. In wild type mice, supernumerary stereocilia can still be found at the front of all hair bundles. At E18.5, mutant hair bundles were indistinguishable from those of controls and all of them showed a staircase-like organization of the stereocilia within middle and basal turns of the cochlea. Some slight degree of misalignment of hair bundle orientation was observed in all three genotypes at E18.5 but this is normal and there is no indication of a planar cell polarity defect at this age. Surprisingly, by P1, hair bundles of wild type littermate controls (Figure 3A–D) were easily distinguishable from those of Tailchaser heterozygotes (Figure 3 E–H) and homozygotes (Figure 3I–L). Both homozygotes and heterozygotes for this mutation show disorganization of hair bundles, reflected in a loss of the characteristic ‘V’ hair bundle shape and an overall flattening or even a concave-like shape of the bundle compared with the characteristic convex ‘V’ shape. In addition, when we measured the width of the P1 stereocilia from the tallest row, we noticed that the wild type stereocilia have a classic Gaussian normal distribution with 0.3 micron average (n = 80), whereas the width of the tallest *Tlc/Tlc* stereocilia distributes in a wide width range with 0.38 micron average (n = 80) (Figure S1). Finally, the hair bundle disorganization is accompanied by a variable position of the kinocilium. To further evaluate the position of the kinocilium we labeled cochlea from P1 wild type, *Tlc/+* and *Tlc/Tlc* mice with an antibody for acetylated tubulin, which stains the kinocilium, and with phalloidin to stain filamentous actin (Figure 3D, H, L). While the kinocilia of the P1 wild type hair cells were uniformly localized to the center of the lateral (strial) side of the hair cell, the kinocilia of the *Tlc/+* mice were dispersed at varying positions between the lateral side of the hair cells and the center of the hair cell. In order to quantify this phenomenon, a region spanning 17–20 hair cells was randomly selected from the basal turn of wild type, *Tlc/+* and *Tlc/Tlc* mice. The location of the kinocilia in reference to the apical surface of the cell was then plotted and quantified as percentage distance from the center of the apical surface of the cell both in the X (apical to basal) and Y (modiolar to striolar) dimensions, with 0% representing the center of the cell and 100% representing the cell surface perimeter (Figure 3M). While in wild type mice the kinocilia were localized to 65% of the distance from the center of the cell to its perimeter in the Y axis (SD 13.22) and 8.6% (SD 14.7) towards the base in the X axis, kinocilia localization in the *Tlc/+* mice was 28% (SD20.48) and −5.25% (28%) in the Y and X axis respectively and −7.6% (SD18.38) and 5.9% (SD 14.6) in the Y and X axis of the *Tlc/Tlc* mice. A t-test analysis shows that while the differences in the localization of the kinocilia along the X-axis of the hair cells is not significantly different between the different genotypes (p values >0.05), the localization of the kinocilia in the Y axis is significantly different between the wild type and *Tlc/+* mice (p value 1.17E-07), wild type and *Tlc/Tlc* mice (p-value 5.09E-15) and between the *Tlc/+* and *Tlc/Tlc* mice (p value 3.24E-06). Interestingly, the average localization of kinocilia of the *Tlc/Tlc* mice was centralized and even closer to the modiolar side of the hair cell apical surface than to the lateral side of the hair cells' apical surface.

### The Disorganization of Tailchaser Bundles Does Not Affect Interstereocilial Links

**Figure 4:**
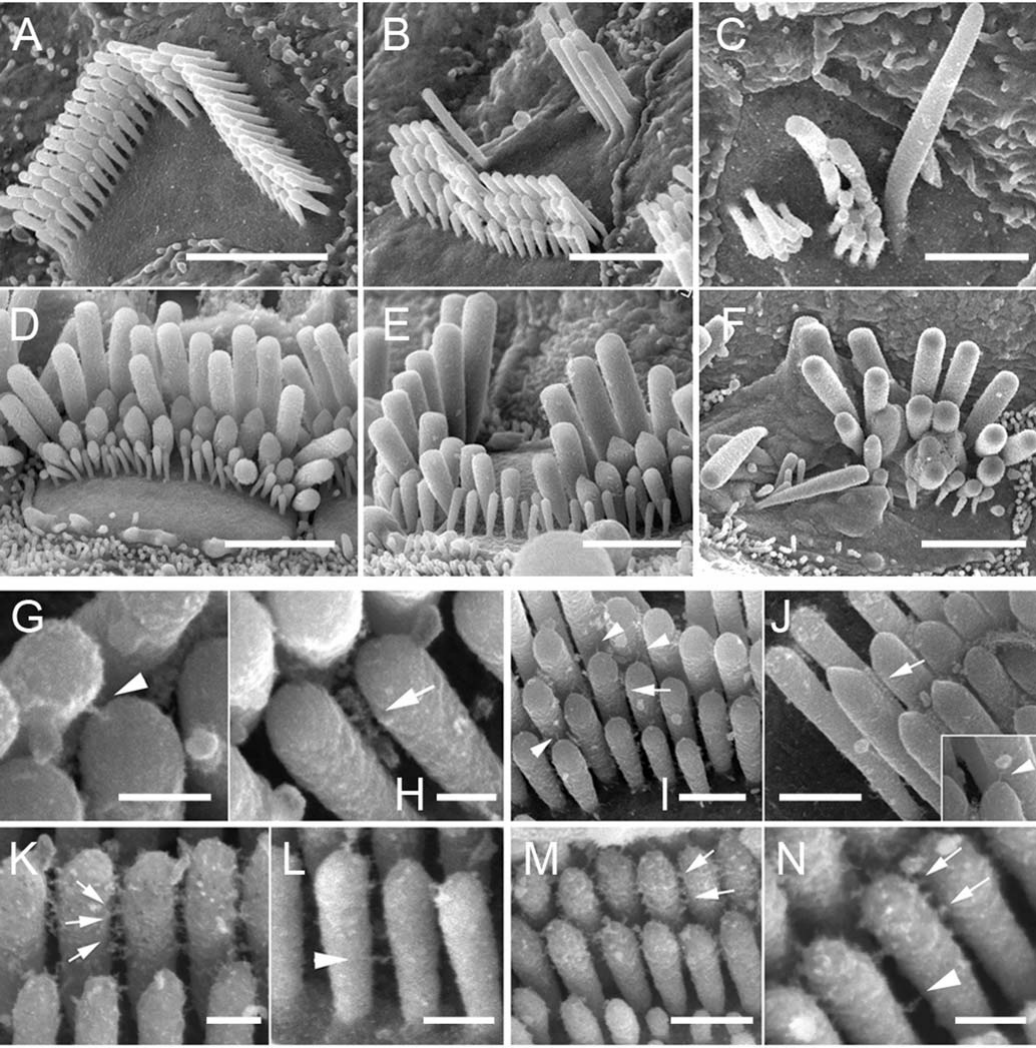
The Tailchaser mutation does not affect formation of interstereocilial links. High resolution SEM images showing stereocilia bundles on outer (A–C, G–N) and inner (D–F) hair cells of wild type (A, D, G, H, K and L), *Tlc/+* (B, E, I, M) and *Tlc/Tlc* (C, F, J and N) mice at P21 (G–J) and P7 (K–N). Note misshaped but staircase-like stereocilia bundles on hair cells of *Tlc/+* animals (B and E) and disorganized bundles formed from fewer stereocilia of *Tlc/Tlc* mice (C and F). The horizontal connectors, tip links (G–J, tip links indicated by arrowheads, horizontal connectors indicated by arrows, inset on J shows high magnification image of tip link found between *Tlc/Tlc* stereocilia), lateral and ankle links (K–N, lateral links indicated by arrows, ankle links indicated by arrowheads) are present between *Tlc/+* and *Tlc/Tlc* stereocilia and are indistinguishable from those of wild type mice. Scale bars: A–F-2 µm; G, H, K, L, N–200 nm; I, J and M–500 nm.

At postnatal day 21 (P21) morphologically mature hair bundles on the apical surface of outer and inner hair cells of wild type mice show a characteristic staircase-like organization of stereocilia graded in height (Figure 4A, D). At this stage of hair bundle development both tip links (Figure 4G) (connecting the pointed tips of shorter stereocilia with the side of adjacent longer stereocilia) and horizontal top connectors (Figure 4H) (forming links along the stereocilia length) were clearly visible on both types of hair cells. As previously described in Tailchaser heterozygotes [27], the stereocilia on outer hair cells form staircase-like bundles but of highly variable shape (Figure 4B). In contrast, in Tailchaser homozygotes, the staircase-like arrangement of stereocilia of graded height was very unclear and hair bundles were disorganized (Figure 4C). Despite severe changes in bundle shape, both tip links and horizontal top connectors were visible in Tailchaser homozygotes and heterozygotes (Figure 4I, J). The morphological changes observed were less pronounced in inner hair cell bundles that still showed a staircase-like stereocilia organization in both hetero- (Figure 4E) and homozygotes (Figure 4F) for the Tailchaser allele. To check if the observed disorganization of the stereocilia in mature hair bundles was caused by an earlier effect of the Tailchaser mutation on the presence or structure of transient interstereocilial links, we analyzed mouse cochleae at P7, expecting both lateral and ankle links to be fully developed by then [32]. At P7, in hair bundles of wild type littermates, lateral links form irregular rays of fine fibers between the upper two thirds of the length of adjacent stereocilia within and between rows and are clearly visible in all bundles analyzed (Figure 4K), while ankle links connecting the basal third of the stereocilia length were seen only sporadically, probably due to the tissue processing method used (Figure 4L). Interestingly, both lateral and ankle links were also present in hetero- (Figure 4M) and homozygous (Figure 4N) Tailchaser mutants and they appeared normal in structure.

### Mutations of Myosin VI Cause Stereocilia fusion and Formation of Stereocilial Branches

**Figure 5:**
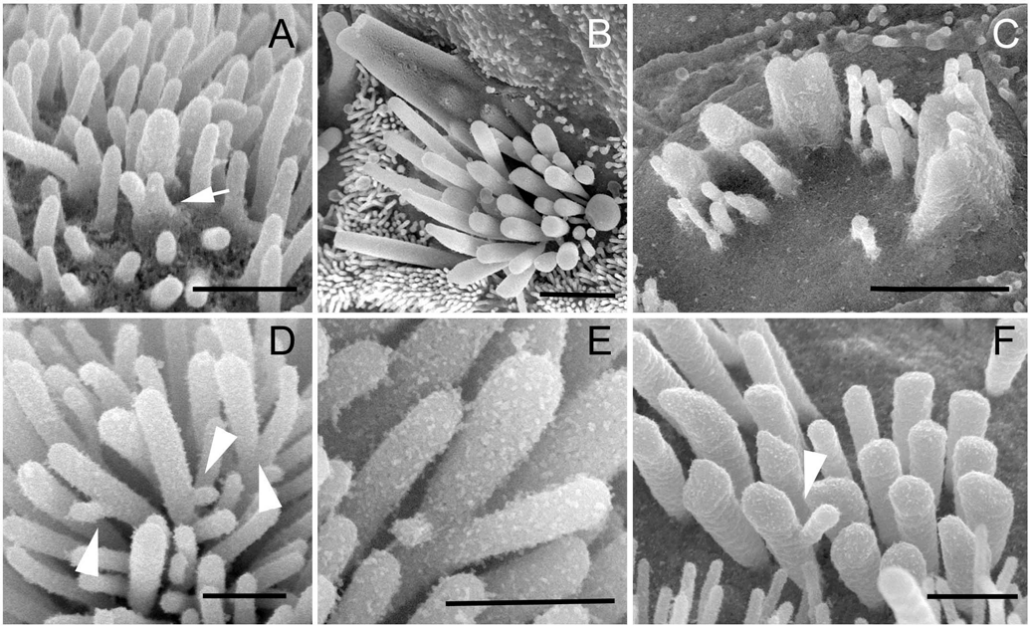
Stereocilium branching is observed in myosin VI mouse mutant hair bundles. (A–C) High magnification SEM images showing stereocilia fusion in developing outer hair cells of *Tlc/Tlc* mice at P1 (A, apical turn) and inner (B, apical turn) and outer (C, basal turn) hair cells of *Tlc/Tlc* mice at P21. Note that stereocilia fusion starts at base of the stereocilia. (D–F) SEM images showing unusual branches found on Tailchaser stereocilia at P1 (D and E) and Snell's waltzer stereocilia at P6. Scale bars: A, D–F–500 nm; B and C–2 µm.

The original null mutation of the *Myo6* gene causes extensive and early stereocilia fusion in Snell's waltzer mice mutants [23]. Tailchaser homozygotes also exhibit stereocilia fusion, which can be observed first at P1 in the apical turn (Figure 5A). At P21 stereocilia fusion was spread along the entire length of the cochlea affecting both outer and inner hair cell bundles in the apical turn (Figure 5B) and mostly outer hair cells in middle and basal turns of the cochlear duct (Figure 5C).

In addition, high-resolution analyses of hair bundles from the apical turn of the cochlea of Tailchaser homozygotes at P1 revealed stereocilia branching (Figure 5D,E), a feature also found in Snell's waltzer mutants [23]. In this study we examined Snell's waltzer mice at P6, when degeneration is quite advanced in the middle and basal turns but mild in the apical region of the cochlea. We confirmed the presence of stereocilia branching in many outer and inner hair cell bundles along the entire length of the cochlea (Figure 5F).

### The Tailchaser Mutation Disrupts Myosin VI's Function in the Transport of Uncoated Endocytic Vesicles

Myosin VI functions in epithelial cells to transport nascent uncoated endocytic vesicles through actin-dense regions [33]–[35]. Disruption of myosin VI activity blocks this process, providing us with an assay to ask whether the D179Y mutation is sufficient to disrupt myosin VI function. We introduced the D179Y point mutation into wild type human myosin VI tagged with Green Fluorescent protein (GFP) [35]. The incorporation of this mutation into the construct, HGFP-M6(D179Y), was confirmed by DNA sequencing and the proper expression was confirmed by Western blot (data not shown).

**Figure 6:**
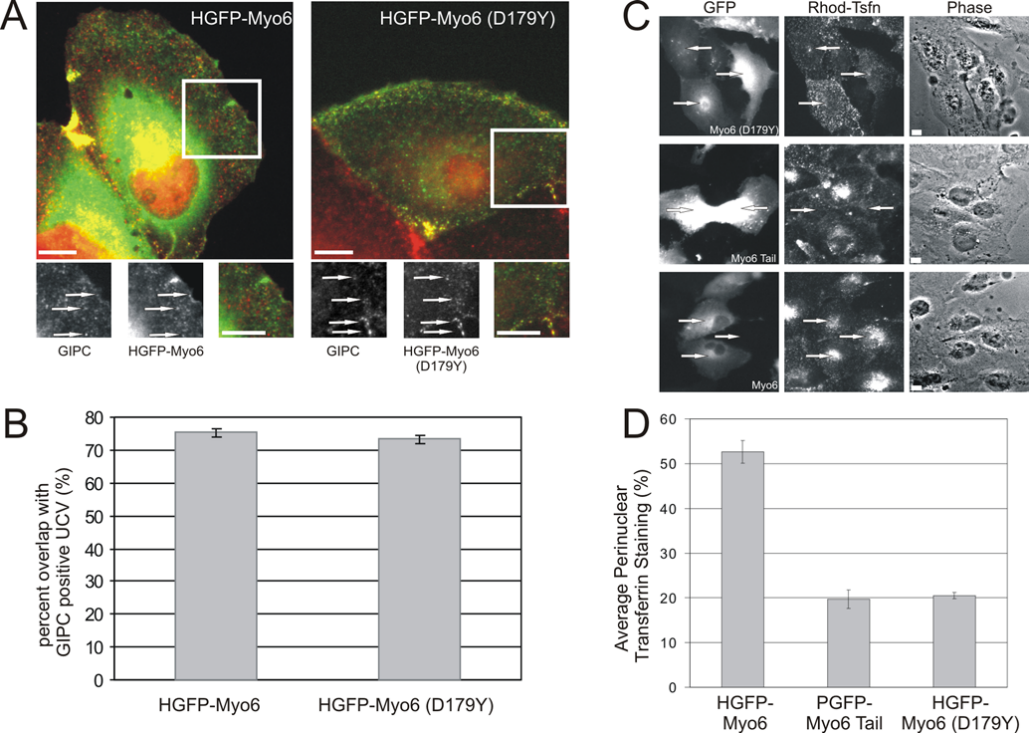
The D179Y mutation disrupts myosin VI's function as an endocytic motor. (A) HGFP-Myo6 (D179Y) is recruited to GIPC-associated uncoated endocytic vesicles. ARPE-19 cells expressing either HGFP-Myo6 or HGFP-Myo6(D179Y) (green) were stained for GIPC (red). Both HGFP-Myo6 and HGFP-Myo6(D179Y) targeted to GIPC-associated uncoated endocytic vesicles (arrows). (B) Quantification showing percent overlap of HGFP-Myo6 and HGFP-Myo6(D179Y) with GIPC-associated uncoated endocytic vesicles. (C) Expression of GFP-Myo6(D179Y) inhibits delivery of endocytic vesicles to the early endosome. Cells were transfected with HGFP-Myo6(D179Y), PGFP-Myo6Tail (a dominant negative construct), or wild type HGFP-Myo6 and allowed to endocytose Rhodamin-conjugated Transferrin (Rhod-Tsfn) for 15 min. (D) Cells transfected with HGFP-Myo6(D179Y), PGFP-Myo6Tail, or HGFP-Myo6 were scored for delivery of Rhod-Tsfn to the early endosome. n = 300 cells and reflects an average of three experiments. Scale bars: 10 µm.

Since the tail of myosin VI is sufficient to target to nascent uncoated vesicles (UCV), we hypothesized that HGFP-M6(D179Y) would also target properly to these vesicles. ARPE-19 cells were transfected with HGFP-M6(D179Y) and then fixed and stained with antibodies specific for GIPC, an adapter protein that collocates with myosin-VI on the UCV surface [33],[35] (Figure 6A). HGFP-M6(D179Y) targeted to peripherally located vesicles with 73% of GIPC-associated UCV colocalizing with the myosin VI protein (Figure 6A, B). Control experiments (not shown) established no overlap between HGFP-M6(D179Y) and clathrin, consistent with the identity of the GIPC and myosin-VI-labeled structures as UCV [33],[34].

Mutations that disrupt myosin-VI motor activity block delivery of UCV vesicles to the early endosomes [33],[34]. This block can be easily visualized by following steady-state uptake of rhodamine-conjugated transferrin (R-Tsfn) [33],[34]. We therefore predicted that overexpression of HGFP-M6(D179Y) would alter R-Tsfn uptake. ARPE-19 cells were transfected with HGFP-M6(D179Y), HGFP-M6 (which has no effect on trafficking [33]), or PGFP-M6tail (which lacks a myosin-VI motor domain and disrupts trafficking [33]), then incubated with R-Tsfn for 15 minutes. Following fixation, the transfected cells were scored for accumulation of transferrin in the perinuclear recycling and early endosome compartment (Figure 6C, D). After 15 minutes of incubation, 52.7+/−2.5% of HGFP-M6 cells transfected exhibited a prominent perinuclear accumulation of R-Tsfn (Figure 6C, D). In contrast, overexpression of GFP-M6(D179Y) caused a drastic decrease in delivery of R-Tsfn to the early endosome with only 20.5+/−0.7% of cells exhibiting a perinuclear accumulation (Figure 6C, D). These results are equivalent to those seen for cells expressing GFP-M6tail (19.7+−2.0% of cells exhibit endosomal delivery), confirming that introduction of the D179Y mutation is sufficient to disrupt myosin VI's function as an endocytic motor.

### The D179Y Mutation Destroys Gating through Disruption of the Transducer Region

**Figure 7:**
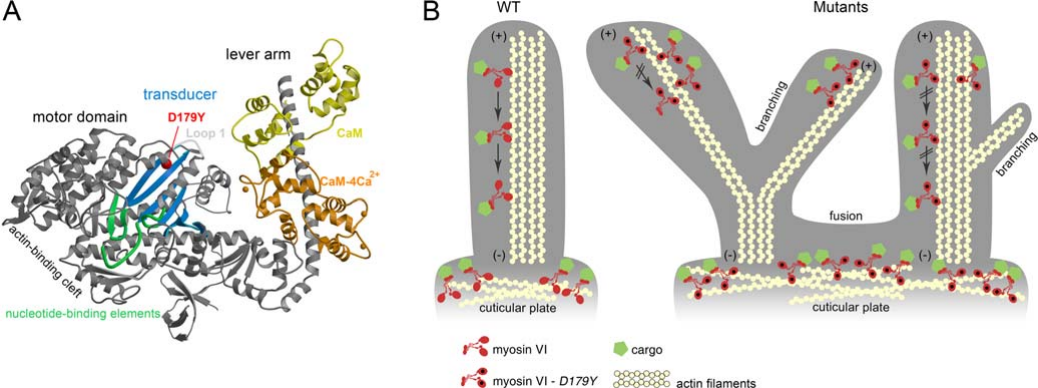
The D179Y mutation in Tailchaser impairs myosin motor function in hair cell stereocilia. (A) The structure of the myosin VI motor domain. The D179Y residue is shown as a red ball and is in the region of a helix that follows Loop 1, the transducer region. (B) Schematic diagram of a wild type and Tailchaser stereocilia demonstrating myosin VI localization and effects of the D179Y mutation on morphology of the base and along the stereocilia. Myosin VI moves along actin by coordinating its heads during processive movement in a process called gating. In Tailchaser, the heads are improperly coordinated, as the D179Y mutation in the two heads are free to independently go through the ATPase cycle without influence of the other head (i.e. the rate per head is identical for the monomer and the dimer). Myosin VI will thus remain in the tips of the stereocilia and subsequently this will result in the inability of myosin VI to transport cargos and/or anchor the membrane of stereocilia to actin filaments, causing single stereocilia to develop branches or to fuse with neighboring stereocilia due to membrane elevation. Alternatively, myosin VI may bind to other structures such as membrane components, leading to the fusion phenotype. Such a region of fusion is demonstrated, as is seen in *sv/sv* P1 mice [23], as well as in Tailchaser in this report. These structural defects impair the hair bundle organization, preventing auditory and vestibular functions.

The D179Y mutation is in a helix that follows a loop (Loop 1) that is involved in altering the steady state ATPase rate and ADP release rate of myosins [36], and precedes the nucleotide-binding element known as switch I [37] (Figure 7A). It is thus in a position to alter communication in the region that has been termed the transducer in myosin motors, which rearranges as myosin goes through its force producing cycle on actin.

To evaluate if the D179Y mutation may disrupt the transducer region, we assessed the maximal steady state actin-activated ATPase rates, which for wild type dimers shows half the rate per head than does a monomer, since the lead head cannot cycle until the rear head detaches. As shown in Table 1, the steady state ATPase rates (actin-activated) for a myosin VI monomer (S1) incorporating the D179Y mutation is decreased compared to wild type. However note that in the mutant dimer, the rate per head is the same as the monomer. Thus gating, or communication between the heads of the dimer, has been destroyed and the mutant myosin is incapable of performing its cell biological functions.

**Table 1:** Actin-activated ATPase activity.

	M6WT(S1^1^)	M6WT (HMM^2^)	D179Y(S1)	D179Y(HMM)
*V*max (+actin) (s^−1^)	7.7	3.5	1.7	1.5
*K*ATPase (µM)	8.1	1.8	1.6	2.0

1 S1-single-headed.

2 HMM–double-headed.

## Discussion

We have identified a *Myo6* mutation affecting the motor domain that permits normal protein expression levels and sub-cellular localization, but disrupts its function as an endocytic motor and prevents appropriate coordination of the myosin heads, known as ‘gating’, to move along actin. Eventually, this leads to disorganized hair cell bundles and branching of stereocilia in mammalian inner ear hair cells. The identified mutation is a G694T transversion leading to an aspartic acid to tyrosine amino acid substitution at position 179, adding to the catalogue of *Myo6* mutations that cause deafness and balance defects in mice and humans.

There are several lines of evidence that implicate the D179Y mutation as being responsible for the Tailchaser phenotype. First, genetic mapping placed the *Tlc* mutation in the same chromosomal interval as *Myo6* and all *Tlc* mutant mice identified by their abnormal behavior carry the G694T mutation, showing that the mutation is present in mice with the mutant phenotype. Furthermore, D179 is evolutionarily conserved. Second, the stereocilia fusion seen in *Tlc* mutant hair cells is very similar to that described in Snell's waltzer myosin VI-null mice. The branching seen for the first time in *Tlc* mutants was also found to be present in Snell's waltzer hair cells. Third, a functional assay revealed that both wild type and a D179Y mutant myosin VI was able to target to nascent uncoated vesicles, but the mutant D179Y myosin VI blocked delivery of UCV vesicles to the early endosomes, similar to the behavior of a motorless myosin VI. Fourth, the location of the D179Y mutation suggests that it would affect the transducer region of the myosin and indeed, measurements of actin-activated ATPase rates demonstrated that the disruption of the transducer region by D179Y leads to loss of ‘gating’, or coordination of myosin VI in dimer form to move along actin. Thus, the D179Y mutation appears to impair the motor function of myosin VI.

Two new features were found in the Tailchaser mutants that confer additional functions for myosin VI in the inner ear. First, myosin VI is expressed specifically in the inner ear hair cells early in development, beginning at E13.5 [22], yet in mutant form, now identified in more than one mouse mutant, hair cells remain indistinguishable from wild type hair cells up to at least E18.5. Detailed SEM analyses of auditory sensory epithelia of homozygous mice revealed that the Tailchaser mutation affects the overall organization of the stereociliary bundle. However, the mutation does not influence the formation of interstereocilial links or planar cell polarity of embryonic hair cells. The stereociliary bundles of outer hair cells in Tailchaser homozygotes progressively lose their staircase-like arrangement but despite these severe morphological rearrangements the formation and maintenance of interstereocilial links is not affected. This indicates that myosin VI is not required for the initial polarization of the hair cells or the proper positioning of the cilia of the bundle or the kinocilium. However, it is necessary for maintenance of the bundle orientation and overall morphology once the bundle has formed and is maturing, possibly via interaction with the cuticular plate. The first steps in hair bundle-formation are marked by the appearance of a central kinocilium (a microtubule-based true cilium) around mouse E13 on the microvilli covered hair cell. Many microvilli eventually develop into stereocilia. By birth, the kinocilium relocates to the middle of the lateral side on the apex of the hair cell, and the ‘V’ shaped hair bundles are all uniformly oriented. The kinocilia of the P1 *Tlc/+* mice are clearly mislocalized and more centrally and variably distributed than the wild type kinocilia, indicating an early defect in their migration towards the lateral pole of the cell. Interestingly, the center of the hair cell bundle seems to follow the kinocilium and the overall organization of the stereociliary bundles in the P1 *Tlc* mutant mice is more flat or concave.

Second, the *Tlc* mutation in myosin VI causes stereocilium branching. The presence of the branching was confirmed in Snell's waltzer, another *Myo6* mutant. This phenomenon occurs at the same time as changes of stereocilia dimensions and suggests myosin VI involvement in not only dynamics of stereocilia actin but also its role in maintenance of parallel actin bundles. These findings are consistent with localization of myosin VI between the actin paracrystal and plasma membrane of stereocilia [30]. The processing myosin VI dimer could transiently interact with both the plasma membrane and actin paracrystal on its way towards the minus ends of the actin filaments localized at the stereocilia bases, and thus create a force that would push the actin paracrystal towards the stereocilia tip. In the absence of functional myosin VI, the forces upon the actin paracrystal and the putative weaker linkage of the plasma membrane to the actin could lead to dysregulation of actin treadmilling in stereocilia, affecting in turn the staircase-like organization of the hair bundle. It remains unclear, however, how presence or absence of myosin VI would influence the stereocilia actin core, leading to formation of stereocilia branches. Myosin VI could create tension between the plasma membrane and actin paracrystal and in this way mechanically inhibit branching. Myosin VI could also interact with the ARP2/3 complex, a complex of seven proteins that binds actin filaments and nucleates new actin filament assembly [38], inhibiting actin filament nucleation and preventing formation of unwanted branches in normal stereocilia. Indeed, in *Drosophila*, myosin VI and the Arp2/3 complex colocalize, suggesting that myosin VI is concentrated in a region of active actin assembly [39].

In this work, we show myosin VI-specific staining along the length of control stereocilia at the level of immunofluorescence. Our current results are consistent with myosin VI immunogold localization [30] and immunofluorescence [40]. Others previously described myosin VI-specific immunofluorescence only in the cell cytoplasm, cuticular plate and stereocilia base [29]. Data obtained by Belyansteva and colleagues using gene gun transfections with Myo6-GFP [41] are consistent with the lack of stereocilia staining as well. The discrepancy between the negative results obtained in 1997 and the current positive stereocilia staining may be explained by differences in sensitivity of the detection systems used. There may be a number of reasons for false negative results of immunohistochemistry and expression of a fluorescently tagged protein (e.g. tag effect on protein function, cell response to mechanical damage). Despite the inherent uncertainty in interpreting negative staining and expression data we do need to consider the possibility that the discrepancies in stereocilia localization that we describe here and previously [30],[41] are false positive results for immunofluorescence and immunogold staining. However, the fundamental novel finding described in this manuscript is that the lack of functional myosin VI predominantly affects the shape and integrity of stereocilia bundles (Figures 3 and 4), indicating that myosin VI plays a role in stereocilia maintenance. The phenotypes that we observe are therefore consistent with stereocilial expression of myosin VI.

For myosin VI to perform its anchoring and processive trafficking functions, it must be able to gate its heads [3]. In the case of myosin VI, the mechanism of gating, or communication between the heads of the dimer, during processive movement involves the lead head being unable to bind ATP until the rear head has released from actin [3]. During anchoring, the lead head would be unable to bind ATP [3], and ADP would tend to out-compete ATP for binding to the rear head [42], preventing either head from releasing from actin. Thus the simplest prediction of why the D179Y mutation, which is in the region of a helix that follows Loop 1 (Figure 7A), causes deafness is that disruption of the transducer region of the myosin destroys gating, and thus allows ATP to bind and dissociate both the lead and rear heads simultaneously. This is easily assessed by the maximal steady state actin-activated ATPase rates, which for wild type dimers shows half the rate per head than does a monomer, since the lead head cannot cycle until the rear head detaches. In the mutant, the actin-activated rate per head is the same in the monomer and dimer, indicative of a loss of gating. Most compelling, the consequence of this immobility renders myosin VI to remain ‘stuck’ at the tips of the stereocilia, rather than expressed along the length, as demonstrated by immunofluoresence studies in *Tlc/Tlc* mutant inner ears.

The presence of myosin VI along the length of the stereocilia in wild type mice, while it is known to move along actin towards the minus end of actin filaments (towards the base of stereocilia), suggests that myosin VI molecules must be transported to the tip by another mechanism not involving the myosin VI motor. Myosin VI could then proceed down the actin filaments using its own motor in wild types, but in the *Tlc* mutants the defective motor would not permit this movement. This hypothesis was supported by our observation of an accumulation of myosin VI labeling at the tips of *Tlc* stereocilia.

How does the loss of myosin VI as a processive motor, either due to its inability to move along actin properly, in the case of Tailchaser, or its absence in the case of Snell's waltzer, translate into the pathology seen in myosin VI-mutated hair cells? The myosin VI motor spends a significant portion of its catalytic cycle bound to actin with a high duty ratio and requires mechanical coordination between the dimer heads while ‘walking’ along actin in relatively large steps [43]. This coordination and communication, or gating, between the two heads is essential. The kinetics of ATP hydrolysis is linked to the length of time each dimer head is attached to actin, determining whether myosin VI will act as a transporter or anchor. When myosin VI cargo is anchored in the membrane, then this motor would serve to apply force on the actin filament to remain close to the membrane, as may be the case in the cuticular plate at the base of the stereocilia (Figure 7B). Lack of myosin VI, or mutant myosin VI that no longer can serve as this anchor, would permit the membrane to detach from the actin filament and the consequence would be fusion of two stereocilia at the base (Figure 7B). A similar mechanism could be taking place that would allow branching of stereocilia to be formed in the absence (Snell's waltzer) or mutant form of myosin VI (Tailchaser) (Figure 7B). This anchoring in the normal state may be achieved, for example, in part by the Arp2/3 complex (described above). Cell adhesion, or lack of it, might be responsible for branching and/or stereocilia fusion due to altered coordination between myosin VI and vinculin and cadherin complexes [20]. While a multitude of cargoes have been identified for myosin VI that bind to its tail region, including GIPC, Ddab2, Sap97, and opineurin [15]–[18], their role in clathrin-coated pits and vesicles in receptor-mediated endocytosis and Golgi secretion has been studied only in epithelial cells. Their task, however, in the inner ear, remains to be elucidated. The identification of the cargos in the stereocilia and cuticular plate may hold the key to understanding how mutant myosin VI causes hair bundle structural changes and ultimately, loss of auditory and vestibular function.

## Materials and Methods

### Mice

The founder mouse carrying the *Tlc* mutation was generated in a large-scale ENU mutagenesis program [26]. All procedures involving animals met the guidelines described in the *National Institutes of Health Guide for the Care and Use of Laboratory Animals,* were approved by the Animal Care and Use Committee of Tel Aviv University (M-00-65) and were in compliance with UK Home Office regulations. The colony was maintained on the original C3HeB/FeJ genetic background.

### Chromosomal Mapping and Sequence Analysis

*Tlc*/+ (C3H) males were outcrossed to wild type C57BL/6 (C57) females in order to generate F1 mutants. F1 mice were phenotyped and identified as mutant if they displayed a strong mutant phenotype that consisted of an impaired reaching response, head bobbing, hyperactivity and severely compromised performance in a swimming test [27]. Phenotyping was performed by two lab colleagues independently. A total of 183 N2 mutants were produced from the two backcross mating protocols, consisting of 46 mutants from the C57 backcross, and 137 mutants from the C3H backcross. The N2 mutant mice from the C57 backcross presented a milder mutant phenotype when compared to the N2 mice of the C3H backcross, resulting in fewer identified mutant mice than would be predicted by Mendelian inheritance.

Genomic DNA was analyzed with MapPairs Mouse microsatellite markers (Invitrogen, http://www.invitrogen.com/), developed at the Whitehead Institute/MIT Genome Center. Fifty-nine markers, polymorphic between C57 and C3H and evenly distributed over the entire mouse genome, were used for a low resolution genome scan using DNA from 21 randomly selected N2 (C3H) mice (Table S1). PCR products were electrophoresed on a 4% MetaPhor (FMC BioProducts, http://www.fmc.com/) gel. After linkage to chromosome 9 was discovered, linkage was further confirmed by typing all N2 mutant mice. Finally, additional chromosome 9 markers were selected for high resolution mapping using N2 mutant mice with informative recombination breakpoints in chromosome 9 (Table S1).

High and low resolution genome scan results were analyzed using ‘Pattern,’ a PERL-based computer software developed for this purpose, designed to facilitate analyzing mouse genome scan results by generating a graphic representation of haplotypes and predicting potential linkage. Pattern uses PCR genome scan results as an input and generates graphic haplotypes and predicts potential linkage to specific genomic intervals. Potential linkage is assigned to a chromosomal marker (available at http://www.tau.ac.il/karena/pattern.html). For each adjacent pair of markers (vertically coupled table analysis), if the number of linked pairs (at least one linked marker out of the pair) through all the patterns is at least 5 times higher than the number of unlinked pairs (both markers unlinked), potential linkage is indicated. For *Tlc* mapping, genome scan haplotypes were then carefully read and the chromosome of linkage, chromosome 9, was identified.

Brain RNA was isolated using RNeasy Mini kit (Qiagen, http://www1.qiagen.com/) and samples were treated with DNAase for removal of genomic DNA, according to the manufacturer's protocol. Mutation analysis was carried out by sequencing of PCR products of *Myo6* cDNA or genomic DNA. Primers available as Supplementary Material (Table S2).

### Genotyping of *Tlc* Mice

A genotyping assay was developed specifically to identify the *Tlc* mutation. The G694 nucleotide resides in a sequence that mimics the *Bcl*I restriction enzyme recognition site with only one mismatch. Genomic DNA was amplified with a reverse primer that contains a 3′ mismatch to artificially create a *Bcl*I restriction enzyme recognition site in the wild type DNA. The *Tlc* G694T mutation eliminates this newly formed restriction enzyme recognition site. Digesting the PCR product with *Bcl*I generates a single band of 118 bp in *Tlc/Tlc* mice, two bands of 32 bp and 86 bp in wild type mice and three bands of 118, 86 and 32 bp in *Tlc/+* mice. Forward primer 5′-CAATATTATTGTTATTCAAGGATTTTTTTTG-3′, reverse primer 5′ AAATTAACAATACCTTCAACAATTCTATG3′.

### Computational Modeling of the Motor Domain of Myosin VI

In order to obtain an extensive multiple sequence alignment, known myosin VI proteins were used to build a hidden Markov model (HMM) profile (using the HMMER package, version 2.0; http://hmmer.janelia.org/; [44],[45], which was then used to search for similar sequences in the SWISSPROT database (Release 48; http://www.expasy.ch/cgi-bin/sprot-search-ful; [46]). Thereafter, redundant sequences were removed using a 90% identity threshold limit. The resulting 77 sequences were multiply-aligned using MUSCLE [47] (http://phylogenomics.berkeley.edu/cgi-bin/muscle/input_muscle.py). The multiple sequence alignment was then used as input to the ConSeq and ConSurf web-servers [48]–[50] (http://conseq.tau.ac.il/ and http://consurf.tau.ac.il/).

Since the mouse myosin VI structure has not been solved yet, the *NEST* program [51] was used to create a homology model. The structure used as template was that of the head domain of porcine myosin VI (PDB ID: *2bkh*) [52]. Since the porcine myosin VI protein shares high sequence similarity (89% identity) with the mouse myosin VI head region, it is likely to be structurally similar and serves as a potentially good template.

### Inner Ear Sensory Epithelium Immunofluorescence

Immunofluorescence of whole mount cochleae harvested from 3 wild type mice at P6, 3 wild type mice at P40 and 8 P70 Tailchaser mice (4 homozygotes and 4 littermate heterozygotes) was performed as described [53]. For protein detection, samples were incubated with a myosin VI antibody at a dilution of 1∶400 (Proteus Biosciences, http://www.proteus-biosciences.com/), and a monoclonal anti α-tubulin antibody (mouse) at a dilution of 1∶200 (Sigma-Aldrich, http://www.sigmaaldrich.com/). DAPI (4′,6-Diamidine-2′-phenylindole dihydrochloride, Roche Applied Science, http://www.roche-applied-science.com/) was used to stain the nuclei in a working solution of 2mg/ml dissolved in water. The secondary antibody (Alexa 488, 1∶1000) and rhodamine conjugated phalloidin were from Invitrogen-Molecular Probes. Images were acquired with a Zeiss LSM510 META confocal microscope, equipped with 63x 1.4NA objective, and processed with LSM Image Browser Rel. 4.2 and Adobe Photoshop CS2. Pixel intensity analyses were performed using Image J software on images acquired at the same settings of the microscope, with background subtracted. Statistical analyses were performed with Xcel software, using the two-tailed test.

### Cell Culture and Transfection

ARPE-19 cells [54] were grown at 37°C with 5% CO_2_ in DMEM-F12 with 10% FBS, fungizone and glutamine and transfected with GFP-tagged myosin- VI constructs as described [33].

### Myosin VI GFP Constructs

HGFP-M6(D179Y) was created using GFP-M6+LI (full length human myosin-VI containing both the small and large tail domain splice insertions fused to GFP [35] using the Quick Change XL Site-Directed Mutagenesis Kit (Stratagene, http://www.stratagene.com/). PGFP-M6tail is a dominant negative construct containing the tail domain of porcine myosin-VI fused to GFP [33].

The primers used were as follows: Forward: 5′-GGAACAGGTCAAGATATTTATGACAGAATTGTTGAAGC-3′ and Reverse: 3′-GCTTCAACAATTCTGTCATAAATATCTTGACCTGTTCC-5′. The mutation in the primer set is underlined. Isolated clones were sequenced to verify that the point mutation was incorporated and that no other mutations were introduced by PCR.

### Cell Culture Immunofluorescence

Coverslip grown cells were processed for immunofluorescence in six-well plates [55]. Affinity-purified rabbit anti-GIPC domain antibodies were used as described [35]. All fixed samples were observed with a Leica DMR upright light microscope fitted with a Hamamatsu ORCA 10bit CCD Digital Camera [33]. Plots and statistical analyses of vesicle properties were generated with Microsoft Excel 2000.

### Pulse-Chase and Steady-State Uptake Assay for Endocytosis

Pulse-chase and steady-state uptakes of rhodamine-conjugated transferrin were undertaken and quantified as described [33]. Quantification of steady-state R-Tsfn uptake to the pericentriolar endosome and quantification of percent overlap between GFP-tagged constructs, R-Tsfn and endocytic markers was carried out as described [33]. Error bars represent the standard deviation from three experiments.

### Scanning Electron Microscopy (SEM) Analysis

A total of 78 Tailchaser mice including 14 E18.5 (5 littermate controls, 4 homozygotes and 9 heterozygotes), 32 P1 (14 littermate controls, 11 homozygotes and 7 heterozygotes), 8 P7 (2 littermate controls, 1 homozygote and 5 heterozygotes), 24 P21 (8 littermate controls, 8 homozygotes and 8 heterozygotes) and a total of 12 P6 Snell's waltzer mice (6 homozygotes and 6 heterozygotes) were investigated by SEM. Freshly isolated cochleae were locally perfused through oval and round windows with 4% glutaraldehyde in 0.07 M (or 0.1 M for adult cochleae) sodium cacodylate buffer pH 7.4 with 3 mM CaCl_2_ and then fixed for 3 h at RT in the same fixative. Samples were then carefully washed in PBS and processed with the OTOTO method (osmium tetroxide/thiocarbohydrazide) adapted from Hunter-Duvar [56], dehydrated in ethanol, critical point dried (CPD 20, Bal-Ted, http://www.bal-tec.com/), mounted on stubs with conductive paint and viewed with a Hitachi FE S-4800 Scanning Electron Microscope operated at 3–5 kV. Samples were viewed without coating or coated with 2 nm of gold (Sputter coater SCD 050, Bal-Tec). Postacquisition image analyses were performed using Adobe Photoshop CS2 and NIH Image softwares (http://rsb.info.nih.gov/nih-image/).

### Protein Expression and ATPase Assays

Myosin VI “zippered” dimer constructs (with and without the D179Y mutation) were created by truncation at Arg-994 (NP_999186 myosin VI [Sus scrofa]), followed by a leucine zipper (GCN4 [57]) to ensure dimerization. The myosin VI-S1 (monomer) constructs (with and without the D179Y mutation) were created by truncation at amino acid 839. In all cases, a Flag tag was appended to the C-terminus to facilitate purification [36]. These constructs were used to create a baculovirus for expression in SF9 cells [36]. ATPase assays were performed as previously described [58].

## Supporting Information

Figure S1 Tailchaser stereocilia are wider than controls. The stereocilia width was measured on a selection of high-resolution SEM images of inner hair cells of wild type (A) and *Tlc/Tlc* (B) mice at P1. Measurements were taken from the upper part of the stereocilia, from the tallest row, as shown on diagram (C). Results show increased variability of stereocilia width within the same row in Tailchaser homozygotes as compared to controls (D).
(3.91 MB TIF)

Figure S2 Myosin VI antibody is specific. The specificity of anti myosin VI antibodies (Proteus Biosciences, green) was validated on tissues harvested from Snell's waltzer mutant mice at P5 using a standard immunofluorescence protocol (see Materials and Methods). Confocal images of inner ear epithelia of +/*sv* (A, B) and *sv/sv* (C, D) mice showed myosin VI specific staining in hair cells of +/*sv* while hair cells of *sv/sv* were myosin VI-negative. Actin filaments were counterstained using rhodamine/phalloidin. Scale bars: 25 µm.
(1.10 MB TIF)

Table S1 Primers for genotyping between C57Bl/6 and C3H.
(0.05 MB XLS)

Table S2 Myosin VI mouse DNA primers.
(0.03 MB XLS)
