# Supplementary figures and images for: A Myo6 Mutation Destroys Coordination between the Myosin Heads, Revealing New Functions of Myosin VI in the Stereocilia of Mammalian Inner Ear Hair Cells
Source: PLoS Genet. 2008 Oct 3;4(10):e1000207. doi: 10.1371/journal.pgen.1000207 (PMC2543112; doi:10.1371/journal.pgen.1000207)

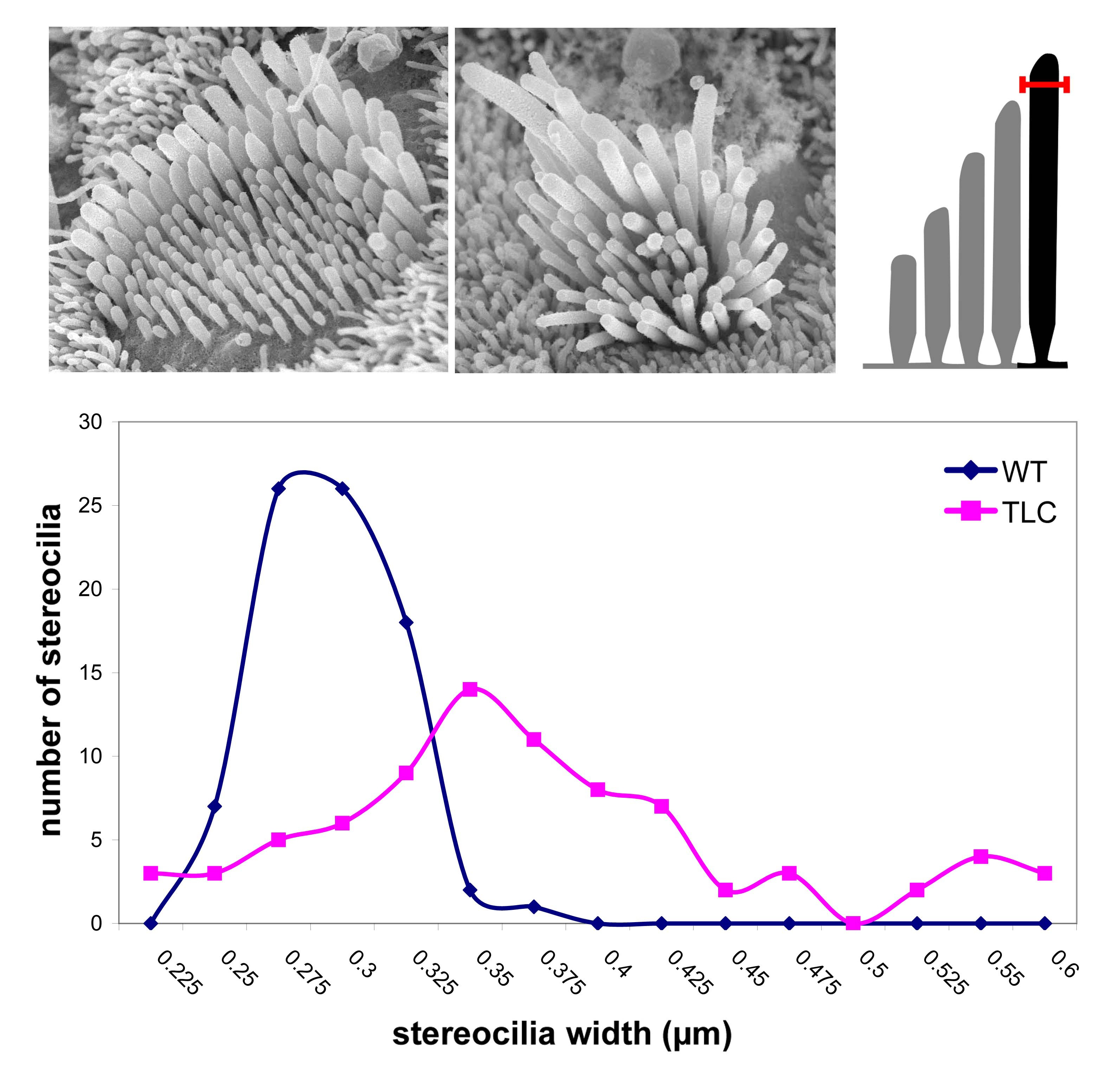

Supplement: Figure S1 — Tailchaser stereocilia are wider than controls. The stereocilia width was measured on a selection of high-resolution SEM images of inner hair cells of wild type (A) and Tlc/Tlc (B) mice at P1. Measurements were taken from the upper part of the stereocilia, from the tallest row, as shown on diagram (C). Results show increased variability of stereocilia width within the same row in Tailchaser homozygotes as compared to controls (D). (3.91 MB TIF) [file pgen.1000207.s001.tif]

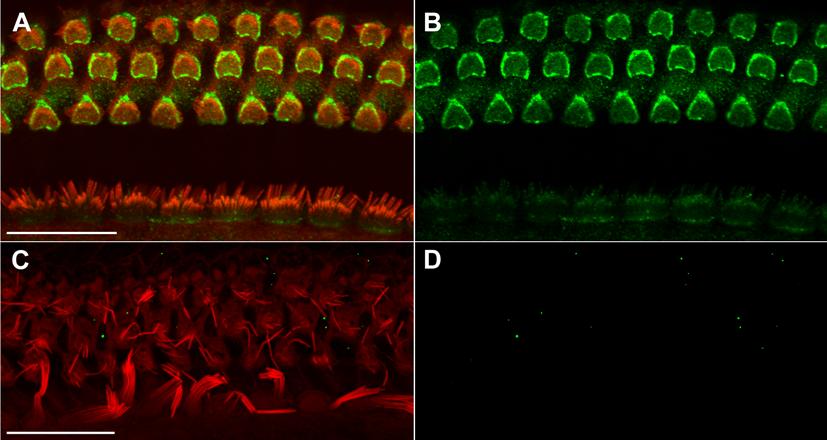

Supplement: Figure S2 — Myosin VI antibody is specific. The specificity of anti myosin VI antibodies (Proteus Biosciences, green) was validated on tissues harvested from Snell's waltzer mutant mice at P5 using a standard immunofluorescence protocol (see Materials and Methods). Confocal images of inner ear epithelia of +/sv (A, B) and sv/sv (C, D) mice showed myosin VI specific staining in hair cells of +/sv while hair cells of sv/sv were myosin VI-negative. Actin filaments were counterstained using rhodamine/phalloidin. Scale bars: 25 µm. (1.10 MB TIF) [file pgen.1000207.s002.tif]
